# Maresin 1 Ameliorates Lung Ischemia/Reperfusion Injury by Suppressing Oxidative Stress via Activation of the Nrf-2-Mediated HO-1 Signaling Pathway

**DOI:** 10.1155/2017/9634803

**Published:** 2017-07-02

**Authors:** Quanchao Sun, You Wu, Feng Zhao, Jianjun Wang

**Affiliations:** ^1^Department of Thoracic Surgery, Union Hospital, Tongji Medical College, Huazhong University of Science and Technology, 1277 JieFang Avenue, Wuhan 430022, China; ^2^Department of Gastrointestinal Surgery, Union Hospital, Tongji Medical College, Huazhong University of Science and Technology, 1277 JieFang Avenue, Wuhan 430022, China

## Abstract

Lung ischemia/reperfusion (I/R) injury occurs in various clinical conditions and heavily damaged lung function. Oxidative stress reaction and antioxidant enzymes play a pivotal role in the etiopathogenesis of lung I/R injury. In the current study, we investigated the impact of Maresin 1 on lung I/R injury and explored the possible mechanism involved in this process. MaR 1 ameliorated I/R-induced lung injury score, wet/dry weight ratio, myeloperoxidase, tumor necrosis factor, bronchoalveolar lavage fluid (BALF) leukocyte count, BALF neutrophil ratio, and pulmonary permeability index levels in lung tissue. MaR 1 significantly reduced ROS, methane dicarboxylic aldehyde, and 15-F2t-isoprostane generation and restored antioxidative enzyme (superoxide dismutase, glutathione peroxidase, and catalase) activities. Administration of MaR 1 improved the expression of nuclear Nrf-2 and cytosolic HO-1 in I/R-treated lung tissue. Furthermore, we also found that the protective effects of MaR 1 on lung tissue injury and oxidative stress were reversed by HO-1 activity inhibitor, Znpp-IX. Nrf-2 transcription factor inhibitor, brusatol, significantly decreased MaR 1-induced nuclear Nrf-2 and cytosolic HO-1 expression. In conclusion, these results indicate that MaR 1 protects against lung I/R injury through suppressing oxidative stress. The mechanism is partially explained by activation of the Nrf-2-mediated HO-1 signaling pathway.

## 1. Introduction

Lung ischemia/reperfusion (I/R) injury occurs in various clinical conditions, such as lung transplantation, pulmonary thrombolysis, and postresuscitation for cardiac arrest, which results in interstitial edema, inflammatory cell infiltration, alveolar-capillary barrier leakage, and gas exchange impairment [1–3]. Particularly in lung transplantation or heart-lung transplantation, I/R is associated with an increased risk of obliterate bronchiolitis (OB), the primary cause of late death following lung transplantation [4]. However, the underlying molecular mechanisms are not fully understood, and ways to prevent lung I/R injury are still lacking.

Many mechanisms, such as sterile immunity, oxidative stress, complement activation, endothelial dysfunction, overactivation of coagulation pathways, and excessive cell death, contribute to the pathogenesis of ischemia/reperfusion [5]. Accumulating experimental evidence proves that the development of oxidative stress induced by the production of reactive oxygen species (ROS) and decreased activity of antioxidant enzyme plays a pivotal role in the etiopathogenesis of I/R. Reactive oxygen species activate subsequent excess leukocyte accumulation and cascades of inflammation, thus causing severe lung damage [6, 7]. Therefore, protecting the lung from oxidative stress could be an ideal method for ameliorating lung I/R injury.

The n-3 polyunsaturated fatty acids (PUFAs) EPA and DHA have been thought to have potential anti-inflammatory and antioxidative stress properties [8]. A recent study highlights the role of endogenous n-3 PUFA-derived anti-inflammatory lipid mediators, such as lipoxins, resolvins, protectins, and maresins, named as specialized proresolving mediators (SPM), in the process of anti-inflammatory, antioxidative, and organ protective processes [9]. Maresin-1 is biosynthesized via lipoxygenase by DHA to generate 14S-hydroperoxydocosa-4Z,7Z,10Z,12E,16Z,19Z-hexaenoic acid, which undergoes further conversion via epoxidation in macrophages and is subsequently converted to 7R,14S-dihydroxydocosa-4Z,8Z,10E,12Z,16Z,19Z-hexaenoic acid, known as maresin 1 (MaR 1) [10]. MaR 1 exerts a number of pharmacological and biological activities and can potentially improve lung performance in multiple conditions. This novel member of n-3 SPM displayed protective effects in zymosan-induced peritonitis, reduced the proinflammatory response of bronchial epithelial cells to organic dust, mitigated LPS-induced lung injury, and alleviated carbon tetrachloride-induced liver injury [11–15]. However, whether MaR 1, the new derivative of n-3 PUFAs, can affect lung I/R injury has not been reported. In the current research, we investigated the impact of MaR 1 on lung I/R injury and explored the possible mechanism involved in this process. Our experiment results revealed that MaR 1 could exert protective effects in lung I/R injury by regulating oxidative and antioxidative levels. To obtain a better understanding of the potential mechanism, we investigated the effects of MaR 1 on Nrf-2/HO-1 signaling pathway in lung I/R injury.

## 2. Materials and Methods

### 2.1. Animals

Specific pathogen free male BALB/c mice, weighing 25–30 g, were purchased from Experimental Animal Center of Tongji Medical College, Huazhong University of Science and Technology. All animals were provided with standard laboratory food and water in a temperature-controlled environment with 12 : 12 hour light/dark cycle, 70% humidity, and 24°C temperature. Animal experimental procedures were approved by the Animal Care and Use Committee of Tongji Medical College of Huazhong University of Science and Technology.

### 2.2. Lung Ischemia and Reperfusion

All animals were anaesthetized with 80 mg/kg of 2% sodium pentobarbital (Sigma-Aldrich, USA) i.p., atropine (Kangqi, Wuhan, China) 0.01 mg/kg i.m, and received anticoagulation with 500 u/kg of heparin sodium (Qianhong, Changzhou, China) i.p 15 min prior to surgery. The animals were mechanically ventilated with a small animal ventilator (ALC-V8, Shanghai, China), adjusting the tidal volume to 2.0–3.0 ml of room air, the respiratory rate to 130–135 per minute, and the inspiratory/expiratory ratio to 1 : 2. The experimental animals were subjected to an occluded left pulmonary hilum with a non-crushing microvascular clamp. After 45 min of ischemia, the clamp was taken away to allow recovery of the blood flow for 150 min, while animals in the sham group underwent thoracotomy without occlusion of the pulmonary hilum [16].

### 2.3. Treatment Protocols

There were six intervention groups, including sham group, MaR 1 group, I/R group, MaR 1 + I/R group, I/R + MaR 1 + Znpp (Znpp-IX, a HO-1 inhibitor) group, and I/R + MaR 1 + Bru (Brusatol, a Nrf-2 antagonist) group, in our experiment. As shown in figures, different groups were chosen according to the experimental objective need. MaR 1 (1.0 ng, Cayman, Michigan, USA) was freshly dissolved in 0.1 ml normal saline (NS). After reperfusion, MaR 1 was administered through the tail vein within 15 min, while the sham group and I/R group received only equal quantities of normal saline. Znpp-IX (10 mg/kg, Sigma-Aldrich Chemical Co, St Louis, USA) was intraperitoneally injected in I/R + MaR 1 + Znpp group 30 min before MaR 1 administration. Brusatol (0.4 mg/kg, BOC Science, Shirley, USA) was given intraperitoneally every other day for a total of five times before surgery in I/R + MaR 1 + Bru group. 150 minutes after reperfusion, all animals were given a lethal injection of sodium pentobarbital. The bronchoalveolar lavage fluid (BALF) was collected by washing the airways of the left lung with 5 ml of phosphate buffer solution through a tracheal catheter (recovery rate > 80%) three times, which was then centrifuged at 3000 rpm/min for 15 min. The left lung was infused with normal saline via the intrapulmonary artery to a pressure of 15 cm H_2_O, the upper part of the left lung was used to measure the W/D weight ratio, and the other portions of the left lung were stored at −80°C or in 10% paraformaldehyde for further use.

### 2.4. Histological Analysis of Lung Tissues

The lower portions of the left lung were fixed in 10% paraformaldehyde for 24 h and then embedded in paraffin. Sections were subsequently stained with haematoxylin and eosin. Lung injury scores were evaluated independently by two pathologists blinded to the treatment groups using the recently published criteria [17].

### 2.5. Quantification of Lung Wet/Dry Weight (W/D) Ratio

To quantify the degree of pulmonary edema, we detected the lung water content. The fresh upper portions of the left lung were wiped-dry with filter paper and then were weighed twice, once before and once after being dried in an oven at 60°C for 48 h. Lung wet/dry weight (W/D) ratio = wet weight/dry weight [16].

### 2.6. Detection of MPO and TNF-*α* Level

Myeloperoxidase (MPO) activity was measured to assess the degree of neutrophil accumulation, an indicator of lung inflammation. Lung tissue samples were harvested and homogenized on ice in five volumes of normal saline. The homogenates were centrifuged at 1200 rpm/min for 10 min. The supernatants were collected and incubated in a water bath for 2 hours at 60°C. Analysis of lung tissue myeloperoxidase was carried out with a commercially available assay kit (Jiancheng Bioengineering Institute, Nanjing, China) according to the manufacturer's instructions. Proinflammatory cytokines are major contributors in lung injury. TNF-*α* level in the supernatant was also measured using commercial enzyme-linked immunosorbent assay (ELISA) kits according to the manufacturer's instructions (Jiancheng Bioengineering Institute, Nanjing, China). Results were standardized for tissue protein concentration. BCA kit was used to detect protein quantification [18].

### 2.7. Determination of BALF Leukocyte Count, BALF Neutrophil Ratio, and Pulmonary Permeability Index

Samples of bronchoalveolar lavage fluid (BALF) were centrifuged (3000 rpm for 5 minutes at 15°C), and then the supernatants were collected. Deposits were used to analyze the number of leukocytes. Through wright staining, the BALF neutrophil ratio was detected. The protein concentration of the BALF supernatant and blood serum were analyzed using a BCA protein assay kit (Pierce Biotechnology, Rockford, IL, USA). Total protein in BALF/total protein in blood serum was calculated, which was given the label Pulmonary Permeability Index (PPI), an indicator of the lung blood-air barrier permeability [19].

### 2.8. ROS, MDA, SOD, GSH-PX, and CAT Activity Assays

Snap-frozen lung tissues were weighed and homogenized in 5 volumes of RIPA buffer on ice. After centrifugation (2000 rpm at 4°C for 10 minutes), the supernatants were collected and stored on ice [15]. The ROS, MDA, superoxide dismutase (SOD), glutathione peroxidase (GSH-PX), and catalase (CAT) activities were measured using their respective commercial assay kits (Jiancheng Bioengineering Institute, Nanjing, China) according to the manufacturers′ instructions. The activities of these indicators were assessed by a spectrophotometer.

### 2.9. Measurement of Lung Free 15-F2t-Isoprostane Level

Free 15-F2t-isoprostane, another specific index of oxidative stress originally from the random oxidation of tissue phospholipids, was detected by using an enzyme immunoassay kit (Cayman chemical, Ann Arbor, MI) as described. Lung tissue homogenates were purified using Affinity Column and Affinity Sorbent (Cayman chemical, Ann Arbor, MI) [20]. The absorbance from the enzymatic reaction was detected at 412 nm, and the values were expressed as pg/g wet protein in tissue homogenates.

### 2.10. Western Blot Analysis

Total proteins were isolated from the lung tissue homogenates according to the protocol provided by the Protein Extraction Reagents Kit (ASPEN, South Africa). Proteins were separated on 10% SDS-PAGE gels by electrophoresis and transferred to a PVDF membrane. The membranes were blocked with 5% nonfat milk for 1 hour and probed with the following antibodies: Nrf-2 antibodies (1 : 200, Santa Cruz, California, USA), HO-1 antibodies (1 : 200, Santa Cruz, California, USA), H2A antibodies (1 : 1000, Santa Cruz, California, USA), and *β*-actin antibodies (1 : 2000, Merck Millipore, Germany). HP-conjugated anti-mouse IgG (Cell Signaling Technology) was used as a secondary antibody. Images were scanned with the canon imaging system and analyzed using Alpha image software.

### 2.11. Statistical Analysis

All experimental data were expressed as mean ± standard error of the mean (SEM) and were analyzed using SPSS software version 17.0 (SPSS Inc., Chicago, IL, USA). Statistical analysis was made using one-way analysis of variance (ANOVA) plus Student-Newman-Keuls post hoc analysis. *P* < 0.05 was considered to indicate a statistically significant difference.

## 3. Results

### 3.1. MaR 1 Ameliorates I/R-Induced Lung Tissue Pathology

**Figure 1:**
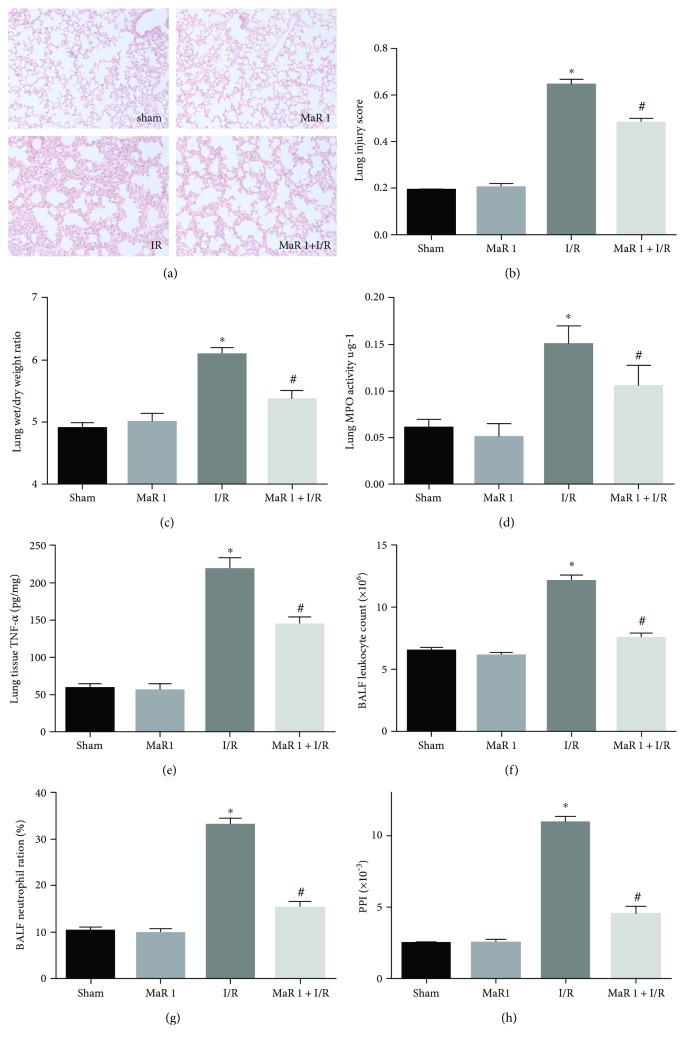
MaR 1 ameliorated ischemia reperfusion-induced lung injury. (a) Representative photographs (200×) showing HE staining of lung sections. (b) Lung injury score. (c) Lung wet/dry weight ration. (d) Lung myeloperoxidase activity. (e) Lung tissue TNF-*α* level. (f) BALF leukocyte count. (g) BALF neutrophil ratio. (h) Pulmonary permeability index (PPI). The results are expressed as the means ± SEM, *n* = 6 per group. ^∗^*P* < 0.05 versus the sham group, ^#^*P* < 0.05 versus the I/R group.

As shown in Figure 1(a), the sham group and MaR 1 group had an approximately normal histological structure with little neutrophil infiltration. In contrast, the I/R group had significantly damaged alveoli structure with interstitial edema, evident inflammatory cell infiltration, and alveolar bleeding. These pathological changes were significantly alleviated in the MaR 1 + I/R group as compared with those seen in the I/R group (*P* < 0.05). Lung injury scores were assessed in parallel with pathohistological changes (Figure 1(b)).

### 3.2. MaR 1 Reduces I/R-Induced Lung Tissue Wet/Dry Weight Ratio, MPO, TNF-*α*, BALF Leukocyte Count, BALF Neutrophil Ratio, and PPI Levels

Figures 1(c), 1(d), 1(e), 1(f), 1(g), and 1(h) show that the values of lung wet/dry weight ratio, MPO, TNF-*α*, BALF leukocyte count, BALF neutrophil ratio, and PPI in the I/R group were significantly higher than those in the sham group and MaR 1 group (*P* < 0.05); whereas, there was no statistic difference between the sham group and MaR 1 group. Compared to the I/R group, these values in MaR 1 + I/R group were significantly reduced (*P* < 0.05).

### 3.3. MaR 1 Suppressed Oxidative Stress and Improved Antioxidant Enzyme Levels during Lung I/R Injury

**Figure 2:**
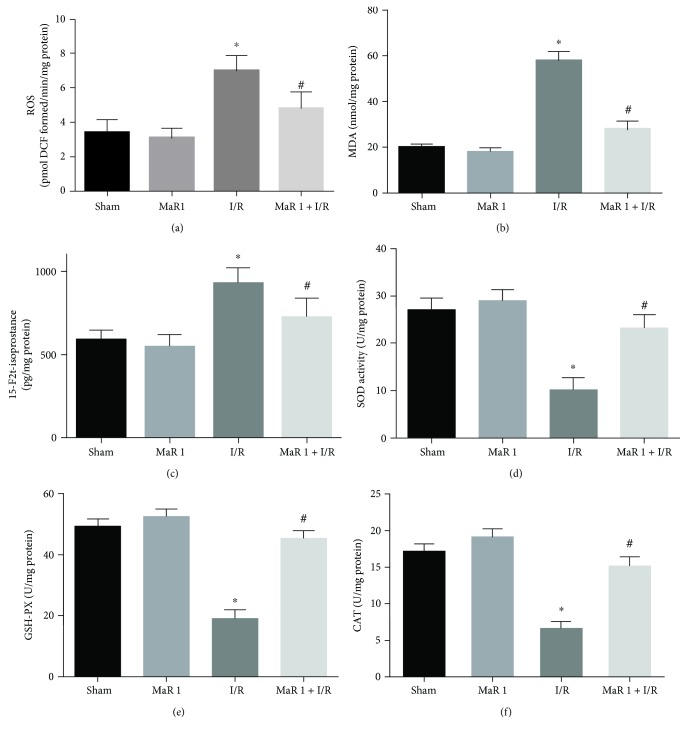
Administration with MaR 1 reduced I/R-induced oxidative stress and restored levels of antioxidant mediators in mice. (a) ROS, (b) MDA, (c) 15-F2t-isoprostane, (d) SOD, (e) GSH-PX, and (f) CAT. The results are expressed as the means ± SEM, *n* = 6 per group. ^∗^*P* < 0.05 versus the sham group, ^#^*P* < 0.05 versus the I/R group.

To further determine the effects of MaR 1 on oxidative stress induced by I/R, the levels of ROS, MDA, and 15-F2t-isoprostane were measured. ROS, MDA, and 15-F2t-isoprostane levels were significantly increased in the I/R group compared with those in the sham and MaR 1 groups (*P* < 0.05). A reduction in ROS, MDA, and 15-F2t-isoprostane formation was observed in the MaR 1 + I/R group as compared with the I/R group (*P* < 0.05), indicating that MaR 1 prevents oxidative stress and lipid peroxidation induced by lung ischemia reperfusion (Figures 2(a), 2(b), and 2(c)). Concomitant to the increased ROS, MDA, and 15-F2t-isoprostane levels, a significant decrease in the activities of antioxidant enzymes (SOD, GSH-PX, and CAT) was detected in the I/R group as compared with that found in the sham and MaR 1 groups (*P* < 0.05). These antioxidant levels were markedly restored under treatment with MaR 1(*P* < 0.05) (Figures 2(d), 2(e), and 2(f)).

These results indicated that MaR 1 likely suppressed I/R-induced oxidative stress via decreasing oxidative stress products (ROS, MDA, and 15-F2t-isoprostane) and increasing the activity of antioxidant enzymes (SOD, GSH-PX, and CAT) in the lung.

### 3.4. Nrf-2/HO-1 Pathway Was Activated by MaR 1 Preconditioning

**Figure 3:**
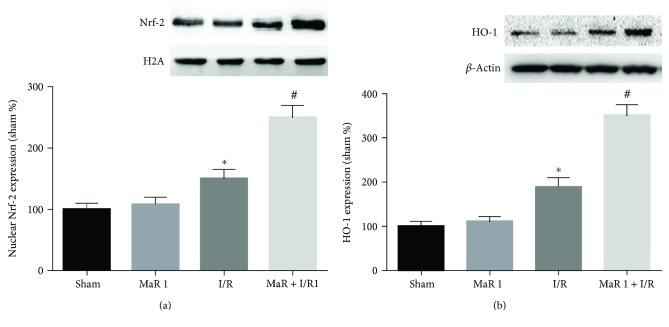
Effects of MaR 1 on Nrf-2/HO-1 pathway. Representative Western blots and quantitative analyses showing nuclear Nrf-2 (a) and cytosolic HO-1 (b) protein expression in the lung. The results are expressed as the means ± SEM, *n* = 6 per group. ^∗^*P* < 0.05 versus the sham group, ^#^*P* < 0.05 versus the I/R group.

The Nrf-2/HO-1 pathway is closely involved in the oxidation and antioxidation processes. The nuclear protein Nrf-2 and cytosolic protein HO-1 were extracted from the lung and analyzed via Western blot analysis. Nuclear Nrf-2 and cytosolic HO-1 were upregulated in the I/R group as compared with the sham and MaR 1 groups (*P* < 0.05). When treated with MaR 1 after lung reperfusion in the MaR 1 + I/R group, the protein expression of nuclear Nrf-2 and cytosolic HO-1 was significantly increased as compared to that of the I/R group (*P* < 0.05) (Figures 3(a) and 3(b)).

### 3.5. ZnPP-IX Administration Suppressed the Protective Effects Elicited by MaR 1 in Lung I/R Injury

**Figure 4:**
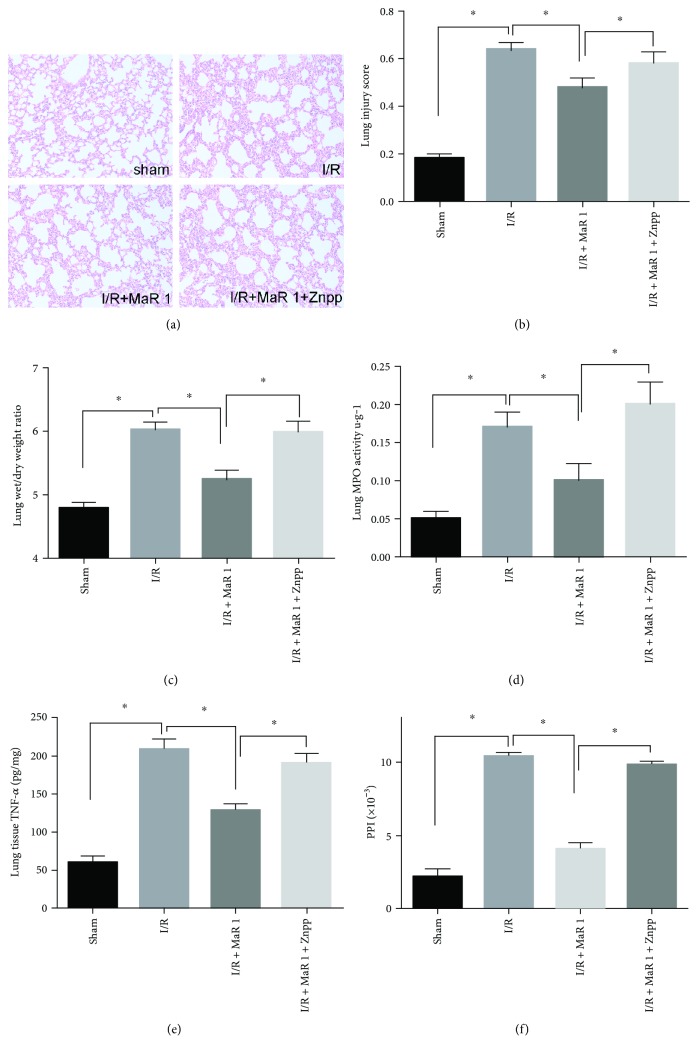
Znpp-IX reversed the protective effects conferred by MaR 1. (a) Representative photographs (200×) showing HE staining of lung sections. (b) Lung injury score. (c) Lung wet/dry weight ratio. (d) Lung myeloperoxidase activity. (e) Lung tissue TNF-*α* level. (f) Pulmonary permeability index (PPI). The results are expressed as the means ± SEM, *n* = 6 per group. ^∗^*P* < 0.05.

To confirm that HO-1 induction is responsible for the protective effects of MaR 1, effects of Znpp-IX, a HO-1 inhibitor, was investigated in lung I/R injury. As shown in Figure 4(a), the I/R group was damaged with interstitial edema, evident inflammatory cell infiltration, and alveolar bleeding. These pathological changes were significantly alleviated in the I/R + MaR1 group. In contrast, after the application of Znpp-IX, the pathological lung injury score became more severe in the I/R + MaR 1 + ZnPP group (*P* < 0.05) (Figure 4(b)).

In agreement with lung tissue pathological changes, lung wet/dry weight ratio, MPO, TNF-*α*, and PPI levels in the I/R group were visibly increased as compared with those seen in the sham group (*P* < 0.05). MaR 1 treatment significantly decreased these changes in the I/R + MaR 1 group, but the protective effect was distinctly blocked by the addition of Znpp-IX, as evidenced by increased lung wet/dry weight ratio, MPO, TNF-*α*, and PPI levels in the I/R + MaR 1 + ZnPP group (*P* < 0.05) (Figures 4(c), 4(d), 4(e), and 4(f)).

### 3.6. Znpp-IX Administration Inhibited the Antioxidant Effects Elicited by MaR 1 in Lung I/R Injury

**Figure 5:**
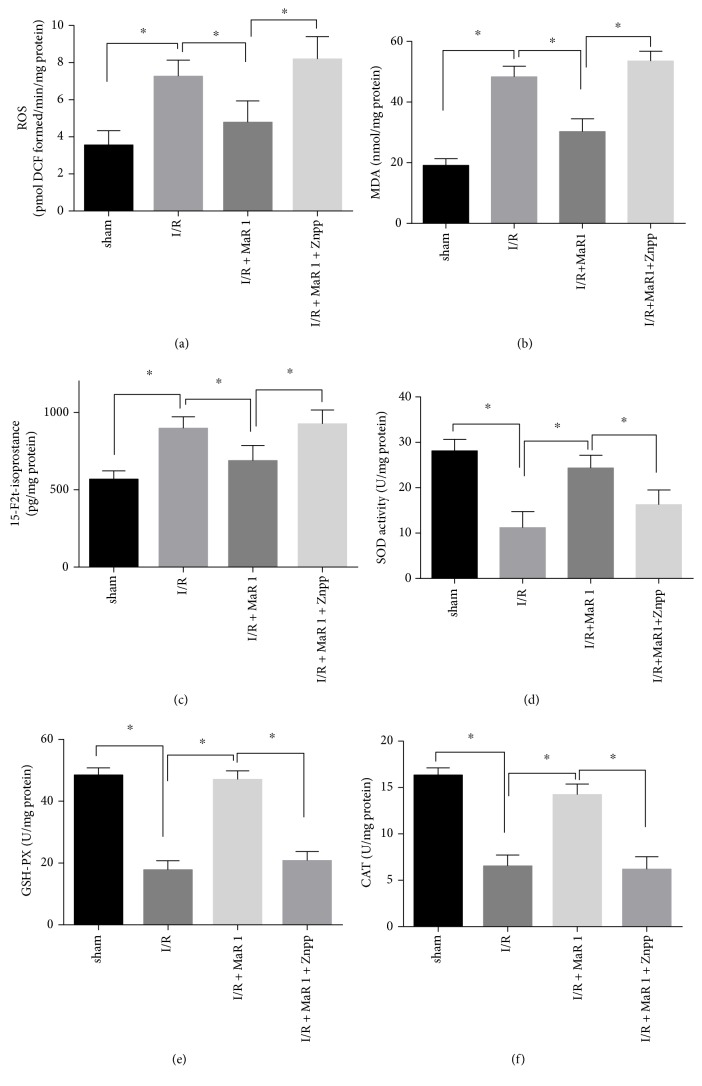
Znpp-IX inhibited the antioxidant effects elicited by MaR 1 in lung I/R injury. (a) ROS, (b) MDA, (c) 15-F2t-isoprostane, (d) SOD, (e) GSH-PX, and (f) CAT. The results are expressed as the means ± SEM, *n* = 6 per group. ^∗^*P* < 0.05.

As for ROS, MDA, and 15-F2t-isoprostane production, the I/R group showed increased values as compared to those seen in the sham and I/R + MaR 1 groups (*P* < 0.05). However, after application of Znpp-IX, ROS, and MDA, 15-F2t-isoprostane production in the I/R + MaR 1 + ZnPP group was significantly elevated as compared to that seen in the I/R + MaR 1 group (*P* < 0.05). Znpp-IX treatment suppressed the antioxidant effects exerted by MaR 1 regarding the changes of ROS, MDA, and 15-F2t-isoprostane levels in lung tissues (Figures 5(a), 5(b), and 5(c)).

Compared to the I/R group, MaR 1 visibly increased the activity of SOD, GSH-PX, and CAT in I/R + MaR 1 group (*P* < 0.05). However, Znpp-IX treatment in the I/R + MaR 1 + ZnPP group caused a significant reduction of these antioxidant enzyme activities as compared to that seen in the I/R + MaR 1 group (*P* < 0.05) (Figures 5(d), 5(e), and 5(f)).

### 3.7. MaR 1 Upregulated HO-1 Expression via Nrf-2 Activation

**Figure 6:**
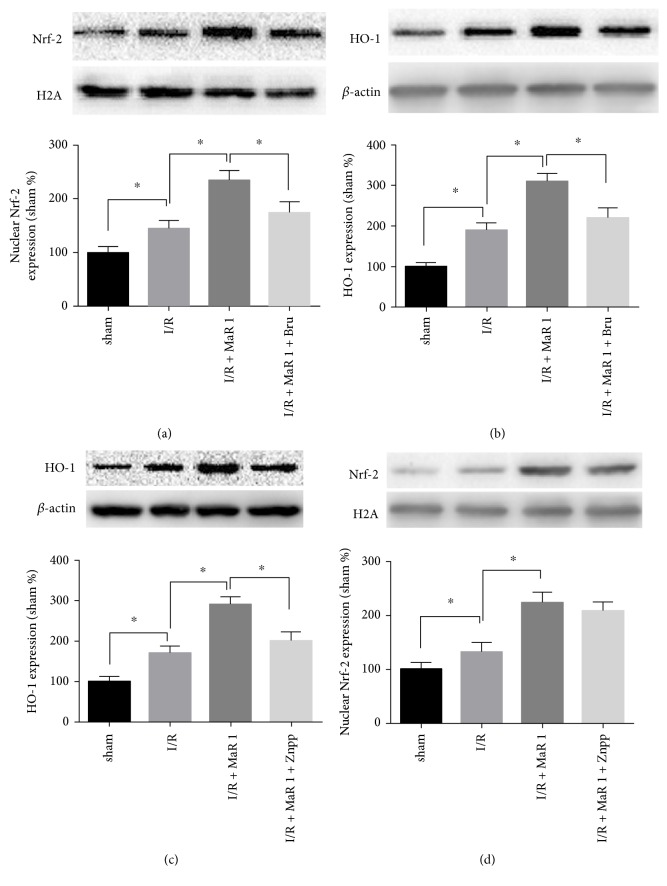
MaR 1 upregulated HO-1 expression may be partly mediated by Nrf-2 activation. Representative Western blots and quantitative analyses showing nuclear Nrf-2 (a) and cytosolic HO-1 (b) protein expression in the lung under treatment of Brusatol. Cytosolic HO-1 (c) and nuclear Nrf-2 (d) protein expression were determined by Western blot in lung under the treatment of Znpp-IX. The results are expressed as the means ± SEM, *n* = 6 per group. ^∗^*P* < 0.05.

It has been known that Nrf-2 plays an important role in the transcriptional regulation of HO-1. Brusatol, an Nrf-2 antagonist, was used to further demonstrate the relationship between MaR 1 and the Nrf-2/HO-1 pathway. After inhibiting Nrf-2 with Brusatol before MaR 1 administration in the I/R + MaR 1 + Bru group, nuclear Nrf-2 protein expression was distinctly decreased (Figure 6(a)), along with reduced cytosolic HO-1 protein expression (Figure 6(b)) compared with that seen in the I/R + MaR 1 group (*P* < 0.05). In addition, compared with the I/R + MaR 1 group, Znpp-IX noticeably inhibited the expression of cytosolic HO-1 induced by MaR 1 in the I/R + MaR 1 + Znpp group (Figure 6(c)), while having no significant influence on nuclear Nrf-2 expression (*P* < 0.05) (Figure 6(d)).

## 4. Discussion

In the current study, we provided evidence that MaR 1 had a protective effect against lung I/R injury. This protective effect likely relied on MaR 1's downstream antioxidative stress capabilities because MaR 1 significantly reduced ROS, MDA, and 15-F2t-isoprostane generation and restored the activity of antioxidative enzymes (SOD, GSH-PX, and CAT). Furthermore, we also found that the protective effects of MaR 1 were inhibited by the HO-1 inhibitor, Znpp-IX, and the expression of HO-1 can be downregulated by Brusatol, an Nrf-2 transcription factor antagonist. This indicated that MaR 1 exhibited its protective effects, at least in part, via the Nrf-2-mediated HO-1 signaling pathway.

The lung structure abnormalities, including destroyed alveoli structure, interstitial edema, inflammatory cell infiltration, and alveolar bleeding were observed in the HE-stained sections of the I/R group. W/D weight ratio, BALF leukocyte count, BALF neutrophil ratio, and PPI level were widely used to indicate the severity of pulmonary epithelial and microvascular permeability [21]. Myeloperoxidase (MPO) accounts for 5% of dry neutrophil cell weight, and the activity of MPO was utilized to indirectly measure the degree of neutrophil infiltration, since it has been proven that reduced neutrophil infiltration protects against lung I/R injury [22]. TNF-*α* is a commonly used inflammatory indicator, which plays an important role in neutrophil-dependent lung injury [23]. All these indices listed above are specific evidence of pulmonary injury and dysfunction; under the treatment of MaR 1, all of these indicators were downregulated, demonstrating that MaR 1 possibly inhibited neutrophil infiltration and the inflammatory response during lung ischemia reperfusion and alleviated lung I/R injury.

Recent reports also indicated that Maresin 1 modulated several innate and adaptive immune cell populations, such as neutrophil, macrophage, and T lymphocyte. Gong et al. proved that Maresin 1 accelerated the resolution of inflammation in LPS-induced acute lung injury through attenuation of neutrophil accumulation, reduction of proinflammatory cytokine production, and acceleration of caspase-dependent neutrophil apoptosis [24]. In vitro, Zhen et al. found that Maresin 1 suppressed LPS-induced proinflammatory cytokine (TNF, IL-1*β*, and IL-8) production and augmented the anti-inflammatory IL-10 release in primary human monocytes/macrophage. They also demonstrated that Maresin 1 inhibited the innate/adaptive bridging, T-cell-stimulating, and angiogenic cytokine, IL-12 p40 [25]. Maresin 1 cannot only directly modulate the inflammatory responses of already existing and activated TH1 and TH17 cells but also can critically prevent their generation from naïve CD4+ T cells acting on their transcription factor-induced activation programs. Additionally, Maresin 1 can enhance the differentiation of CD4+ T cells into Treg cells [26]. Yang et al. demonstrated that Maresin 1 augmented de novo generation of regulatory T cells (Tregs), which interacted with ILC2 (group II innate lymphoid cells) to markedly suppress cytokine production, and accelerated resolution of allergic lung inflammation. Inflammation has been implicated as an important role in ischemia reperfusion injury, and blocking various aspects of the inflammatory cascade has been proved to alleviate ischemia reperfusion injury. In the process of inflammation, innate and adaptive immune cells, neutrophil, macrophage, and CD4+ T lymphocyte accumulate and contribute sequentially in reperfusion, which play a pivotal role in lung ischemia reperfusion injury. The new study found that T lymphocyte cell, which activated and infiltrated into lung during reperfusion earlier than neutrophil, contributes importantly to lung I/R injury [27]. In view of this, we hypothesise that the innate and adaptive immune cells may also be involve in the protective effects conferred by Maresin 1 in lung I/R injury, and this needs to be further investigated in our next study.

Lung ischemia reperfusion injury is a complicated pathophysiological process, in which oxidative stress also plays a critical role [7]. During ischemia, endothelial cells, neutrophils, and macrophages produce ROS, which stimulates nicotinamide adenine dinucleotide phosphate (NADPH), nuclear factor-κB (NF-κB), and proinflammatory cytokines. The increased ROS can also trigger a response from the graft in transplantation, resulting in the activation of the adaptive immune response through the activation of antigen-presenting cells [28, 29]. In addition, the excess generation of ROS, resulting in protein and DNA damage through lipid peroxidation, is believed to be an important cause of oxidative damage to cellular membranes, leading to cell death in lung I/R injury. The level of tissue MDA and 15-F2t-isoprostane, which is the product of lipid peroxidation, was significantly increased in the lung after ischemic reperfusion. These reactions exacerbate the inflammatory response and lung dysfunction, leading to pulmonary edema and gas exchange impedance [20, 30].

The elimination of reactive free radicals is primarily achieved by a series of antioxidant enzymes, such as SOD, GSH-PX, and CAT, which maintains the balance of oxidative stress and antioxidative stress response. The loss of antioxidant enzymes evidently causes free radical accumulation and further aggravates lung I/R injury [31, 32]. Interestingly, a previous study reported the antioxidative activity of MaR 1 in CCl4-induced liver injury [15]. However, there is no research reporting whether MaR 1 could confer a similar protective effect against lung I/R injury. Importantly, Lipoxin A4, another specialized proresolving mediator, was demonstrated to exert an antioxidative effect against intestinal mucosa injury through the Keap1/Nrf-2 pathway following ischemia reperfusion [33]. Zhao et al. reported that resolvin D1 significantly improved SOD and GSH-PX activity and attenuated lipid peroxidation, indicating that the scavenging ability of oxygen free radicals was enhanced by resolvin D1 in lung I/R injury [16]. At present, our in vivo study firstly showed that the formation of ROS, MDA, and 15-F2t-isoprostane induced by lung I/R could be inhibited by administration of MaR 1, another special proresolving lipid. Furthermore, MaR 1 can also restore the activity of antioxidative enzymes (SOD, GSH-PX, and CAT). Therefore, it is reasonable to believe that the protective effect of MaR 1 on lung I/R injury is partially due to the maintenance of balance between oxidative and antioxidative stress.

We also sought to investigate the possible mechanism and signaling pathway underlying the protective effect. The transcription factor Nrf-2, which interacts with antioxidant response elements (AREs), has recently been proved to be a major player in controlling the expression and induction of a battery of defensive genes encoding antioxidant enzymes, such as NADPH quinone oxidoreductase 1 (NQO1), superoxide dismutase (SOD), and heme-oxygenase-1 (HO-1) [34]. Accumulating evidence suggests that the upregulation of genes driven by Nrf-2/AREs protects against myocardial ischemia reperfusion injury [35, 36]. It has also been proven that HO-1 activation performs a fundamental role in endogenous anti-inflammatory and antioxidative stress processes. HO-1 is an inducible enzyme responsible for the conversion of heme to carbon monoxide (CO), iron, and biliverdin. Biliverdin reductase, in turn, converts biliverdin into bilirubin, which has been proven to be a potent antioxidant [37]. Wu et al. reported that hypercapnic acidosis enhanced HO-1 expression and attenuated lung I/R injury; however, HO-1 inhibitor, Znpp-IX, partially inhibited these protective effects [38]. They also demonstrated that valproic acid attenuated lung I/R injury by decreasing lung edema, production of inflammatory cytokines, reactive oxygen species, and apoptosis, whereas valproic acid enhanced HO-1 activity. The protective effect of valproic acid was also mitigated by the presence of Znpp-IX [39]. Yang et al. verified that HO-1, upregulated by pterostilbene, suppressed mitochondrial oxidative stress in vitro and in vivo in an experimental model of cerebral ischemia reperfusion injury [40]. Marcos et al. demonstrated that inhibition of HO-1 or silencing of Nrf-2 blocked the protective effects of Carnosic acid on mitochondria-related redox parameters and function, which explicated a role of the Nrf-2/HO-1 axis in the maintenance of mitochondrial function during oxidative stress [41]. Our present study showed that MaR 1 increased nuclear Nrf-2 and cytosolic HO-1 expression in lung tissue after ischemic reperfusion. Znpp-IX, as a potent HO-1 activity inhibitor, reversed the protective effect of MaR 1 on lung I/R injury, as well as MaR 1's antioxidative stress effects. In addition, the Nrf-2 transcription factor inhibitor, Brusatol, significantly decreased MaR 1-induced nuclear Nrf-2 expression and decreased HO-1 expression, indicating that the induction of HO-1 may be partly mediated by Nrf-2 activation.

In summary, we provided the first piece of evidence that MaR 1 had a protective effect against lung I/R injury. This protection likely relied on antioxidative stress capabilities that were mediated at least in part by the Nrf-2/HO-1 signaling pathway. The results of the investigation help to confirm the protective effect of MaR 1 in lung I/R injury and further increase understanding of its pharmacological effects. However, additional research in which lung endothelial cells transfected with siRNA or plasmid of Nrf-2/HO-1 in the context of an oxygen-glucose deprivation (OGD)/recovery model needs to be conducted in order to verify the present study.
